# Serum peptidomic profiling identifies systemic JAK–STAT and PI3K–Akt pathway dysregulation in cats with chronic gingivostomatitis

**DOI:** 10.1093/jvimsj/aalag169

**Published:** 2026-08-25

**Authors:** Sekkarin Ploypetch, Apisit Pornthummawat, Pruettha Aruvornlop, Sittiruk Roytrakul, Janthima Jaresitthikunchai, Narumon Phaonakrop, Supakit Buamas, Panithi Sukho

**Affiliations:** Department of Clinical Sciences and Public Health, Faculty of Veterinary Science, Mahidol University, Nakorn Pathom 73170, Thailand; Department of Pre-Clinic and Applied Animal Science, Faculty of Veterinary Science, Mahidol University, Nakhon Pathom 73170, Thailand; Department of Pre-Clinic and Applied Animal Science, Faculty of Veterinary Science, Mahidol University, Nakhon Pathom 73170, Thailand; National Center for Genetic Engineering and Biotechnology, National Science and Technology Development Agency, Pathum Thani 12120, Thailand; National Center for Genetic Engineering and Biotechnology, National Science and Technology Development Agency, Pathum Thani 12120, Thailand; National Center for Genetic Engineering and Biotechnology, National Science and Technology Development Agency, Pathum Thani 12120, Thailand; Diagnostic Division, Prasu-Arthorn Animal Hospital, Faculty of Veterinary Science, Mahidol University, Nakhon Pathom 73170, Thailand; Department of Clinical Sciences and Public Health, Faculty of Veterinary Science, Mahidol University, Nakorn Pathom 73170, Thailand; Department of Companion Animal Clinical Sciences, Faculty of Veterinary Medicine, Kasetsart University, Bangkok 10900, Thailand

**Keywords:** cats, chronic gingivostomatitis, peptide biomarkers, peptidome, serum

## Abstract

**Background:**

Chronic gingivostomatitis in cats is a debilitating inflammatory disease marked by severe oral pain and systemic immune dysregulation. Although localized oral pathology is well-documented, the systemic peptidomic profile remains largely unclear.

**Hypothesis/Objectives:**

Characterize the serum peptidomic profiles of cats with feline chronic gingivostomatitis (FCGS) and identify systemic biomarkers and signaling hubs that drive disease pathogenesis and therapeutic interactions.

**Animals:**

Client-owned cats with clinically and histopathologically confirmed FCGS (*n* = 34) and healthy controls (*n* = 18).

**Methods:**

A case–control, cross-sectional study was conducted. All cats were screened for feline leukemia virus, feline immunodeficiency virus, and feline heartworm infections. Serum samples were analyzed using matrix-assisted laser desorption/ionization-time-of-flight mass spectrometry (MALDI-TOF MS) to produce peptide mass fingerprints. Detailed peptide identification was conducted using nanoscale liquid chromatography–tandem mass spectrometry (nano LC–MS/MS). Functional enrichment and protein–protein/chemical interaction networks were developed using ShinyGO and the Kyoto Encyclopedia of Genes and Genomes database.

**Results:**

Both MALDI-TOF MS and nano LC–MS/MS identified distinct systemic protein signatures that distinguished FCGS from controls. Fifteen proteins showed significant changes, with 13 increased (including inflammatory markers such as interleukin (IL)-6 and interferon-γ) and 2 decreased (gastrin, IL-18). Network analysis identified 8 key proteins (eg, p21(CDKN1A), CASP1, IL-6) that interact with FCGS drug treatments. These systemic changes focus on the Janus kinase–signal transducers and activators of transcription (JAK–STAT) and phosphatidylinositol 3-kinase (PI3K)/protein kinase B (AKT; PI3K–Akt) pathways, which are important therapeutic targets.

**Conclusions and clinical importance:**

Our study provides a molecular framework for FCGS. Whereas MALDI-TOF MS enables rapid systemic profiling, nano LC–MS/MS identifies key dysregulated pathways, specifically IL-6/JAK–STAT and PI3K–Akt. These findings offer potential therapeutic targets and a basis for monitoring molecular pathology in affected cats.

## Graphical Abstract

**Graphical Abstract:**
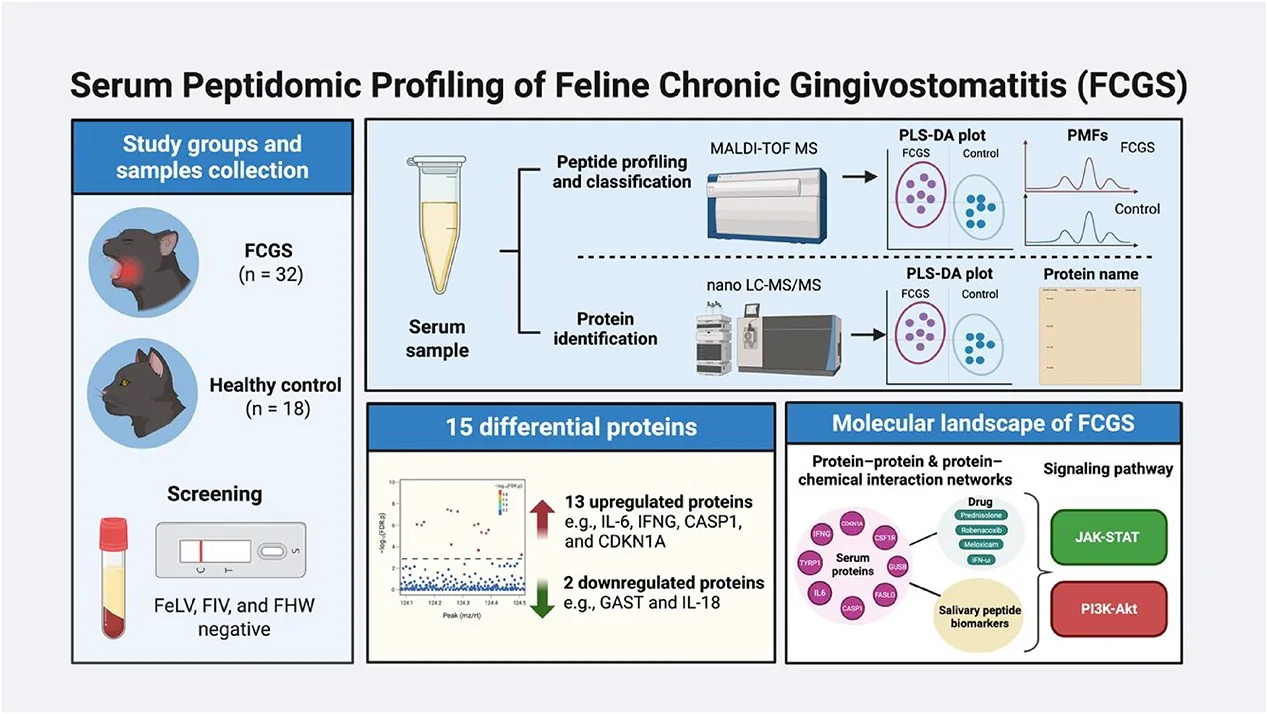

## Introduction

Chronic gingivostomatitis in cats is a severe, debilitating, and intensely painful inflammatory condition of the oral mucosa in cats, most notably in the caudal oral cavity.^1^^,^^2^ This chronic oral disease markedly impairs a cat’s quality of life and often leads to anorexia and weight loss. Histologically, feline chronic gingivostomatitis (FCGS) lesions are defined by a dense lymphoplasmacytic infiltration of the oral mucosa.^3^^,^^4^ The disease is recognized as immune-mediated, but its etiopathogenesis is complex and multifactorial.^2^^,^^5^ Transcriptomic analyses of affected oral tissues consistently identify an inflammatory response markedly influenced by upregulated interleukin-6 (IL6) and signaling pathways such as nuclear factor κ B (NF-κB), Janus kinase/signal transducers and activators of transcription (JAK–STAT) signaling pathway, and interferon (IFN) types I and II.^6^^,^^7^ This systemic-local inflammation is further accompanied by increased circulating concentrations of acute phase proteins such as alpha-1-acid glycoprotein, which correlate with disease severity.^8^^,^^9^ Furthermore, FCGS has been associated with systemic effects, including inflammatory changes in the esophagus and lower respiratory tract, often without obvious clinical signs.^10^ The systemic response in FCGS is characterized by increased circulating CD8+ effector memory T cells and a decreased CD4/CD8 ratio, indicating persistent, unresolved inflammation.^2^^,^^4^

Although surgical treatment remains the gold standard for treatment of FCGS, approximately 30% of cases are refractory and require prolonged medical management.^11^^,^^12^ Given that biopsy currently is used primarily to rule out mimicking lesions rather than to confirm a clinical diagnosis, there is a critical need for non-invasive biomarkers to monitor active molecular pathology and guide therapeutic decisions.^4^ Peptidomics offers a powerful platform for identifying novel circulating inflammatory protein mediators.^13^^,^^14^ Although salivary peptidomic studies in FCGS have identified distinct peptide signatures related to the JAK–STAT and phosphatidylinositol 3-kinase (PI3K)/protein kinase B (AKT) signaling pathway (PI3K-Akt pathway), a similar large-scale investigation using serum, a readily available sample reflecting systemic disease, to comprehensively explore the peptidome, specifically linking to viral status and clinical markers, is critically lacking.^15^

Our research aims to address the current gap in FCGS biomarker knowledge by using serum analysis. We utilized advanced, untargeted proteomic techniques: Matrix-assisted laser desorption/ion-ization-time-of-flight mass spectrometry (MALDI-TOF MS) for rapid initial profiling (peptide mass fingerprinting) and comprehensive nano-liquid chromatography-tandem MS (nano LC–MS/MS) for detailed peptide sequencing and quantification.^15^ By comparing the serum peptidomes of FCGS cats and controls, and considering retroviral status (feline leukemia virus [FeLV] and feline immunodeficiency virus [FIV]), we sought to identify novel systemic biomarkers and core pathological drivers, to provide a molecular foundation for precision therapeutic interventions in FCGS.

## Materials and methods

### Animals and sampling

In our prospective case–control study, 34 cats with caudal stomatitis and histopathologically confirmed FCGS and 18 healthy controls (CTRL) were enrolled. All cats were screened for FIV, FeLV, and feline heartworm (FHW) infections using the SNAP Feline Triple Test (IDEXX). Inclusion criteria for FCGS required severe caudal mucositis and stomatitis. All medications were discontinued 7 days before sampling. Blood was collected under sedation or anesthesia before oral intervention, stabilized with protease inhibitors, and stored at −80 °C. Total protein concentration was measured using the modified Lowry assay. The study was approved by the Mahidol University Animal Care and Use Committee (MUVS-2023-10-64).

### Serum peptide profiling by mass spectrometry

#### Cluster analysis by MALDI-TOF MS

Initial peptide mass fingerprinting (PMF) was performed using a JMS-S3000 SpiralTOF™-plus 2.0. Serum peptides (0.1 μg/μL) were combined with α-cyano-4-hydroxycinnamic acid (CHCA) and analyzed in linear positive mode (1000-6000 Da). Eight replicates per sample were used to create partial least squares-discriminant analysis (PLS-DA) clusters.^14^

#### Peptide identification by nano LC–MS/MS

High-resolution peptide identification was carried out using an Ultimate 3000 Nano LC system coupled to a ZenoTOF 7600 mass spectrometer (SCIEX). Peptides were separated on a 15 cm C18 column with a 30-min gradient ranging from 5% to 55% acetonitrile. The instrument operated in data-dependent acquisition (DDA) mode with Zeno trap activation for MS2 spectra (100-1800 m/z).

### Bioinformatics and statistical analysis

Peptide quantification was managed using MaxQuant (v. 2.5.0.0) against the *Felis catus* UniProt database on November 2, 2025.^16^^,^^17^ Search parameters included unspecific digestion, 0.6 Da mass tolerance, and a 1% protein false discovery rate (FDR). Significantly altered peptides were identified using an FDR-adjusted *P*-value < .05. Statistical and functional interpretations were performed using MetaboAnalyst 6.0 (http://www.metaboanalyst.ca).^18^ Peptides were deemed significantly altered if they concurrently met stringent criteria: FDR-adjusted *P*-value < .05.

### Pathway and interaction analysis

Protein–protein and protein-chemical interaction networks were analyzed using the STITCH and Kyoto Encyclopedia of Genes and Genomes (KEGG) databases.^19^ Functional enrichment was performed using ShinyGo v.0.85 (*F. catus*).^20^ The analysis specifically mapped candidate proteins against common FCGS therapeutics such as nonsteroidal anti-inflammatory drugs (NSAIDs), prednisolone, cyclosporine, interferon-ω (IFN-ω), and oclacitinib. Finally, a targeted interaction analysis was conducted to correlate serum findings with 9 previously identified salivary biomarkers, identifying convergent signaling pathways.^15^

## Results

### Cohort demographics and histological findings

We enrolled 34 cats diagnosed with FCGS and 18 healthy CTRL cats. The FCGS group had a mean age of 6.82 ± 3.3 years, ranging from 2 to 14 years, whereas the CTRL group had a mean age of 7.5 ± 2.26 years, with ages ranging from 5 to 12 years. Detailed demographic information, including breed and sex distribution, is provided in Table 1.

**Table 1:** Shows the results of breeds, sexes, and ages, including the detection of feline immunodeficiency virus (FIV), feline leukemia virus (FeLV), and *Dirofilaria immitis* in serum samples collected from cats diagnosed with feline chronic gingivostomatitis (FCGS), as well as those in the healthy control group.

No.	Histological finding	Groups	Breeds	Sexes	Ages (years)	Immunoassay of FIV, FeLV, and heartworms		
						FIV	FeLV	Heartworms
**1**	Severe necrotic lympho-plasmacytic and suppurative gingivitis	FCGS	DSH	F	3	-	(+)	-
**2**		FCGS	DSH	Fs	5	-	-	-
**3**		FCGS	DSH	Mc	7	-	-	-
**4**		FCGS	DSH	Fs	14	-	-	-
**5**		FCGS	DSH	Fs	8	-	-	-
**6**		FCGS	DSH	Fs	5	-	-	-
**7**		FCGS	DSH	Mc	13	-	-	-
**8**		FCGS	DSH	Mc	11	-	-	-
**9**		FCGS	DSH	Fs	8	(+)	-	-
**10**		FCGS	DSH	Fs	7	-	-	-
**11**		FCGS	DSH	Fs	11	(+)	-	-
**12**		FCGS	DSH	M	4	-	(+)	-
**13**		FCGS	DSH	Fs	11	(+)	-	-
**14**		FCGS	DSH	M	8	-	-	-
**15**		FCGS	DSH	Mc	6	-	-	-
**16**		FCGS	DSH	M	4	-	-	-
**17**		FCGS	DSH	Mc	11	-	-	-
**18**		FCGS	DSH	Fs	5	-	-	-
**19**		FCGS	DSH	Fs	11	-	-	-
**20**		FCGS	DSH	Mc	5	-	-	-
**21**		FCGS	DSH	Mc	4	-	-	-
**22**		FCGS	DSH	Fs	11	-	-	-
**23**		FCGS	DSH	Mc	8	-	-	-
**24**		FCGS	DSH	Fs	5	-	-	-
**25**		FCGS	DSH	Mc	4	-	-	-
**26**		FCGS	DSH	Mc	2	-	-	-
**27**		FCGS	DSH	Fs	4	-	-	-
**28**		FCGS	DSH	Fs	3	-	-	-
**29**		FCGS	DSH	Mc	5	-	-	-
**30**		FCGS	Siamese	Fs	9	-	-	-
**31**		FCGS	Persian	Fs	4	-	-	-
**32**		FCGS	British Short Hair	Fs	2	-	(+)	-
**33**		FCGS	DSH	Fs	7	(+)	-	-
**34**		FCGS	DSH	Mc	9	-	-	-
**35**	Proliferating fibrous connective tissue	CTRL	DSH	Fs	5	-	-	-
**36**		CTRL	DSH	Mc	5	-	-	-
**37**		CTRL	DSH	Mc	6	-	-	-
**38**		CTRL	DSH	Fs	6	-	-	-
**39**		CTRL	DSH	FS	8	(+)	-	-
**40**		CTRL	DSH	Mc	8	-	-	-
**41**		CTRL	DSH	Fs	8	-	-	-
**42**		CTRL	DSH	Fs	9	-	-	-
**43**		CTRL	DSH	Fs	9	-	-	-
**44**		CTRL	DSH	Mc	7	-	-	-
**45**		CTRL	DSH	Fs	5	(+)	(+)	-
**46**		CTRL	DSH	M	9	-	-	-
**47**		CTRL	Siamese	Mc	12	-	-	-
**48**		CTRL	Persian	Fs	12	-	-	-
**49**		CTRL	Persian	Mc	9	-	-	-
**50**		CTRL	American Short Hair	Mc	7	-	-	-
**51**		CTRL	Scottish Fold	Mc	5	-	-	-
**52**		CTRL	Maine Coon	Fs	5	-	-	-

Abbreviations: CTRL = healthy control group; DSH = domestic short hair; F = female; FCGS = feline chronic gingivostomatitis; FeLV = feline leukemia virus; FIV = feline immunodeficiency virus; Fs = female sprayed; M = male; Mc = male castrated.

For cats in the FCGS group, clinical signs consistently lasted more than 6 months. Oral examination identified typical inflammatory lesions, mainly affecting the left and right caudal buccal mucosa. Histologic examination was performed to rule out other oral diseases and consistently showed severe pathology in all FCGS biopsy samples, further supporting the clinical diagnosis. The lesions were characterized by intense inflammation, with infiltration of plasma cells and lymphocytes into the caudal oral submucosa. Notably, neutrophil infiltration was seen in 50% of cases, and Mott cells (B lymphocytes containing immunoglobulins) were often present.^15^ In contrast, histologic analysis of the CTRL group showed only proliferating fibrous tissue and epithelial hyperplasia, consistent with mild mucositis or healthy tissue, and lacked the pronounced inflammation seen in the FCGS group.

### Prevalence of FIV, FeLV, and FHW status

The prevalence of FIV and FeLV was assessed using the SNAP Feline Triple test on 52 serum samples. In the FCGS group, 4 of 34 samples (11.67%) tested positive for FIV (antibodies), and 3 of 34 samples (8.82%) tested positive for FeLV (antigen). The remaining 27 FCGS cats showed no evidence of infection. In the CTRL group, 2 of 18 samples (11.11%) were positive for FIV, and 1 of 18 samples (5.56%) was positive for FeLV, with 1 cat in the CTRL group coinfected with both FIV and FeLV (Table 1). Fisher’s exact test was used for statistical analysis. Comparison of FIV infection frequency (FCGS: 4/34 vs CTRL: 2/18) was not significantly different between the groups (*P* = 1.0). Similarly, the frequency of FeLV infection was not significantly different between the groups (FCGS: 3/34 vs CTRL: 1/18; *P* = 1.0). All cats tested negative for FHW antigen.

### Serum peptide profiling and classification by MALDI-TOF MS

**Figure 1:**
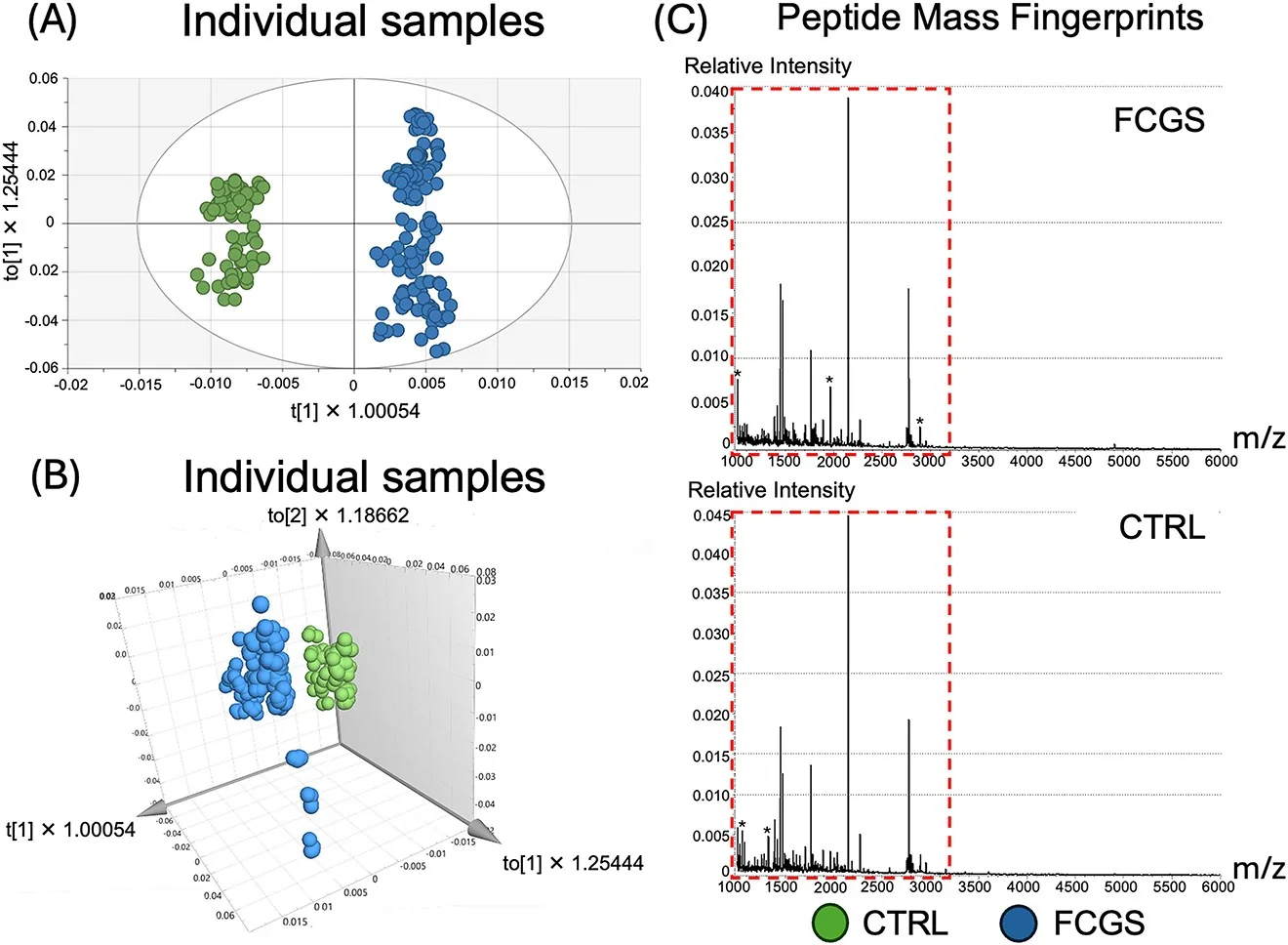
Multivariate analysis and representative MALDI-TOF MS spectra of serum samples. (A) 2D and (B) 3D partial least squares-discriminant analysis (PLS-DA) plots demonstrate distinct spatial clustering between the feline chronic gingivostomatitis (FCGS) group and the healthy control (CTRL) group, with axes labeled by their respective variance components. (C) Representative peptide mass fingerprints (PMFs) from pooled serum samples illustrate systemic peptidomic signatures. The dashed box highlights the 1-6 kDa mass range, where the most prominent spectral differences occur. Asterisks (*) denote specific peptide peaks that effectively discriminate the FCGS peptidome from the CTRL profile.

The initial screening of serum peptides was performed using MALDI-TOF MS to evaluate overall peptidome differences. Spectral analysis confirmed separation of the study groups: PLS-DA plots showed clear, distinct clusters between the FCGS and CTRL groups in both 2D and 3D views (Figure 1A and B). All samples in the CTRL group demonstrated strong profile consistency, but 3 samples from the FCGS group were identified as outliers and excluded from the PLS-DA plots. Comparing the PMFs from pooled samples identified consistent differences in peptide expression, with distinct PMF signals ranging from 1 to 6 kDa between the 2 groups (Figure 1C). These MALDI-TOF MS results clearly demonstrated substantial differences in the serum peptidome and supported the hypothesis that peptide profiles differed between FCGS and CTRL serum samples. As a result, the peptide profiles of all suitable samples were subjected to higher-resolution nano LC–MS/MS analysis to accurately identify potential peptide biomarkers.

### Identification of differentially expressed peptides and proteins by nano LC–MS/MS

**Figure 2:**
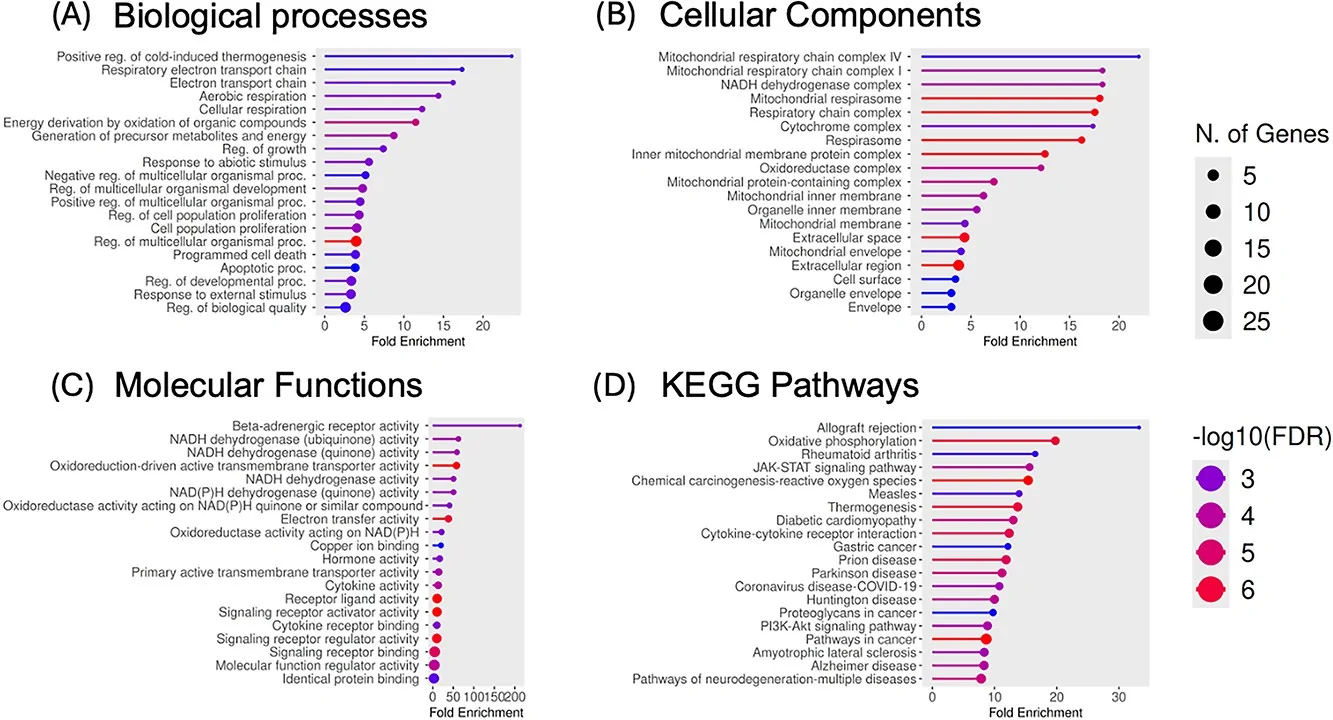
Functional enrichment and pathway analysis of differentially expressed serum peptides in feline chronic gingivostomatitis (FCGS). Functional characterization of identified candidate peptides was performed using ShinyGO v.0.88 and the KEGG database based on the *Felis catus* proteome. The dot plots display significantly enriched terms across 4 categories: (A) biological processes, (B) cellular components, (C) molecular functions, and (D) KEGG pathways. The y-axis shows the specific functional terms, while the x-axis indicates the fold enrichment. The size of each dot represents the number of genes associated with the pathway, and the color gradient reflects the significance level based on the false discovery rate (FDR).

The 233 proteins identified across all samples were analyzed using in-solution digestion and LC–MS/MS (see Table S1). Functional analysis of these serum peptides provided clear molecular evidence of underlying chronic inflammation, oxidative stress, and cellular dysregulation closely associated with FCGS, as illustrated in Figure 2A-D (see also Tables S2-S5). Notably, mitochondrial respirasome markers were detected extracellularly, indicating altered cellular respiration associated with chronic inflammation. The KEGG pathway analysis directly linked the altered peptide profile to chemical carcinogenesis involving reactive oxygen species, oxidative phosphorylation, and major oncogenic signaling (Figure 2D).^21^ Key inflammatory signaling cascades, including the JAK–STAT (FDR: 1.39 × 10^−8^) and PI3K-Akt (FDR: 3.42 × 10^−8^) signaling pathways, were significantly enriched, reinforcing their potential roles in disease development and consistent with previous findings in saliva of affected cats.^15^

**Figure 3:**
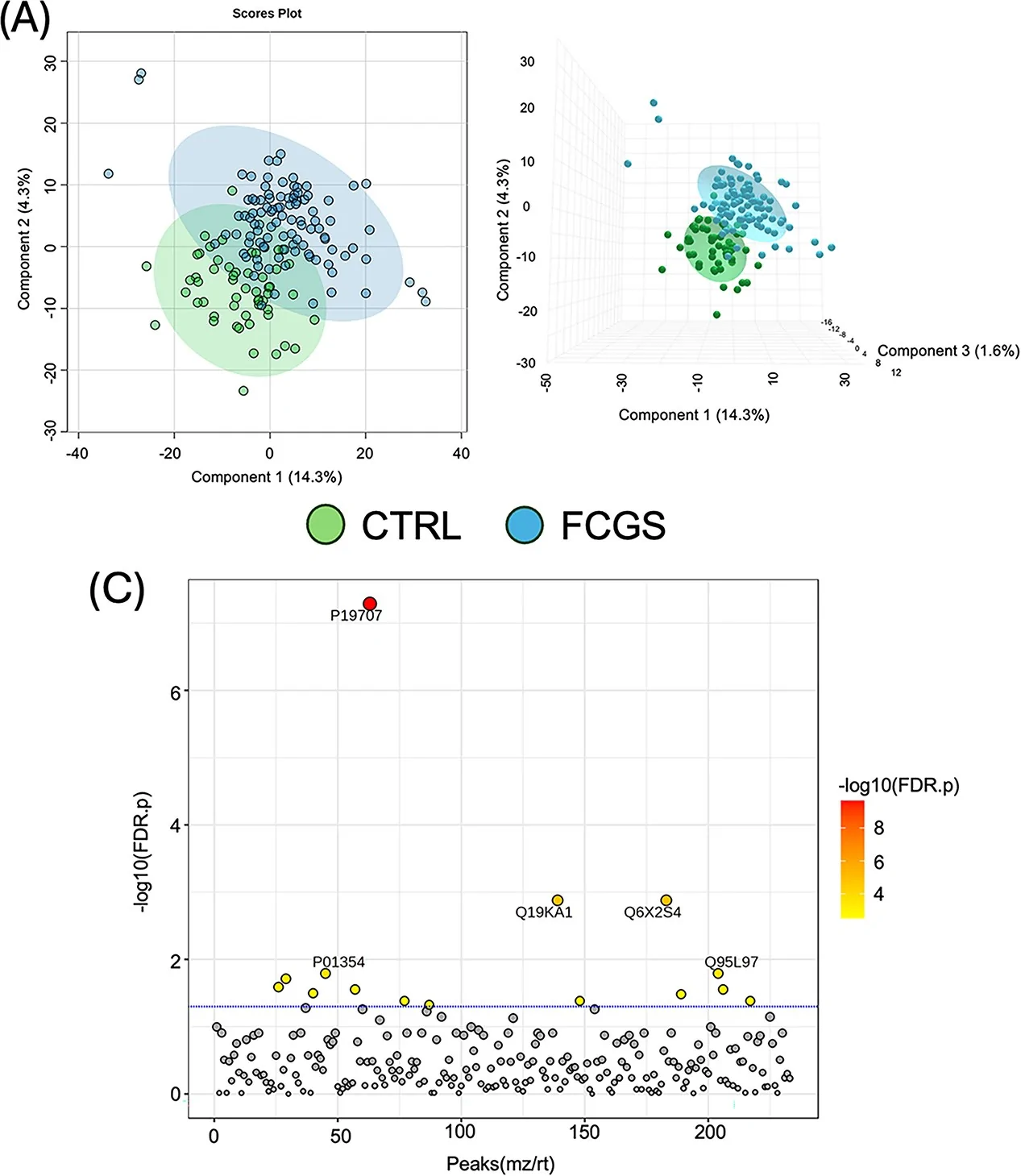
Multivariate and differential expression analysis of serum peptidome profiles via LC–MS/MS. (A) 2D and (B) 3D partial least squares-discriminant analysis (PLS-DA) plots show distinct clustering and spatial separation of serum peptide profiles between cats with feline chronic gingivostomatitis (FCGS) and healthy controls (CTRL). The clear separation validates the high level of peptidomic variation associated with the disease state. (C) Manhattan plot illustrating the differential abundance of serum peptides. Each point represents an individual peptide, with the y-axis showing statistical significance (−lo*g*_10_ false discovery rate (FDR)–adjusted *P*-value). The points are categorized based on their level of statistical significance as defined in the plot key: highly significant peptides (highest −lo*g*_10_ FDR values), peptides with lower significance that still meet the threshold, and peptides that are not statistically significant (*P* ≥ .05).

To validate separation of the study cohorts, we performed PLS-DA, which provided a robust visualization of the peptidomic data from LC–MS/MS. Both 2D and 3D PLS-DA plots identified clear, non-overlapping clusters, confirming that the serum peptide profiles of the FCGS group were distinctly segregated from those of the healthy CTRL cats (Figure 3A and B). Subsequent univariate analysis using a Manhattan plot identified significant differences in peptide abundance based on Wilcoxon rank-sum tests across all detected peaks. Using an FDR-adjusted *P*-value threshold of <.05, 15 peptides were identified as significantly altered (Figure 3C). Among these, 13 proteins were upregulated in the FCGS group including serum amyloid A protein (SAA), inhibin β A chain (INHBA), glucose-6-phosphatase catalytic subunit 1 (G6PC1), sodium-dependent phosphate transporter 2 (SLC20A2)/(PiT-2), cyclin-dependent kinase inhibitor 1 (CDKN1A), major prion protein (PrP), CD antigen CD230, macrophage colony-stimulating factor 1 receptor (CSF1R), β-glucuronidase (GUSB), tumor necrosis factor ligand superfamily member 6 (FASLG), caspase-1 (CASP1), IL-6, tyrosinase-related protein 1 (TYRP1), and interferon γ (IFN-γ). In contrast, 2 proteins, gastrin (GAST) and interleukin-18 (IL-18), were significantly downregulated (Table 2).

**Table 2:** Serum proteins with different expression levels identified by nano LC–MS/MS in cats with FCGS compared to healthy controls.

**Protein IDs**	**Protein names (peptide sequence)**	**Gene name**	**−log10** ***P*-value**	**FC**	**Direction**	**FDR**	**Biological process**	**Cellular component**	**Molecular function**
**P19707**	Serum amyloid A protein (SAA; AAKVISDAREN; AAKVISDARENSQRVT)	SAA1	9.6551	1.15	Up	5.16 × 10^−8^	Acute-phase response [GO:0006953]	High-density lipoprotein particle [GO:0034364]	N/A
**Q6X2S4**	Inhibin β A chain (AALLNAIRK; AALLNAIRKLH)	INHBA	4.8348	1.07	Up	0.0013224	Activin receptor signaling pathway [GO:0032924]; endodermal cell differentiation [GO:0035987]; extrinsic apoptotic signaling pathway [GO:0097191]	Activin A complex [GO:0043509]; enzyme activator complex [GO:0150005]; extracellular region [GO:0005576]	Cytokine activity [GO:0005125]; growth factor activity [GO:0008083]; hormone activity [GO:0005179]
**Q19KA1**	Glucose-6-phosphatase catalytic subunit 1 (AAHFPHQVVA; AAHFPHQVVAGVLSGIAV)	G6PC1	4.7689	1.11	Up	0.0013224	Cholesterol homeostasis [GO:0042632]; gluconeogenesis [GO:0006094]; glucose 6-phosphate metabolic process [GO:0051156]	Endoplasmic reticulum membrane [GO:0005789]; membrane [GO:0016020]	Glucose-6-phosphatase activity [GO:0004346]; phosphate ion binding [GO:0042301]
**Q95L97**	Sodium-dependent phosphate transporter 2 (AADSSSAP; AADSSSAPED)	SLC20A2	3.4606	1.07	Up	0.016134	Phosphate ion transmembrane transport [GO:0035435]; positive regulation of bone mineralization [GO:0030501]	Apical plasma membrane [GO:0016324]; brush border membrane [GO:0031526]; plasma membrane [GO:0005886]	Phosphate transmembrane transporter activity [GO:0005315]; sodium:phosphate symporter activity [GO:0005436]; virus receptor activity [GO:0001618]
**O19002**	Cyclin-dependent kinase inhibitor 1 (AGCVQEARERWNFDFV; AGCVQEARERWNFDFVTETPLE)	CDKN1A	3.3017	1.13	Up	0.019388	Signal transduction by p53 class mediator [GO:0030330]; fibroblast proliferation [GO:0048144]	Cytoplasm [GO:0005737]; cytosol [GO:0005829]; nuclear body [GO:0016604]; nucleolus [GO:0005730]	Cyclin binding [GO:0030332]; cyclin-dependent protein kinase activating kinase activity [GO:0019912]
**P13369**	Macrophage colony-stimulating factor 1 receptor (AAQIVCSASN; AAQIVCSASNI)	CSF1R	2.9666	1.04	Up	0.027959	Inflammatory response [GO:0006954]; innate immune response [GO:0045087]; osteoclast differentiation [GO:0030316]	Cell surface [GO:0009986]; CSF1-CSF1R complex [GO:1990682]; plasma membrane [GO:0005886];	Cytokine binding [GO:0019955]; growth factor binding [GO:0019838]; macrophage colony-stimulating factor receptor activity [GO:0005011]
**O97524**	β-glucuronidase (AAFLLRERYWKLANETRY; AAFLLRERYWKLANETRYP)	GUSB	2.8646	1.04	Up	0.031825	Carbohydrate metabolic process [GO:0005975]	Extracellular space [GO:0005615]; lysosome [GO:0005764]	β-glucuronidase activity [GO:0004566]; carbohydrate binding [GO:0030246];
**Q861W5**	Tumor necrosis factor ligand superfamily member 6 (ADHLYVNVSE; ADHLYVNVSELSLVSFEESKTFF)	FASLG	2.8087	1.09	Up	0.032904	Immune response [GO:0006955]; necroptotic signaling pathway [GO:0097527]; negative regulation of angiogenesis [GO:0016525]	Cytoplasmic vesicle lumen [GO:0060205]; extracellular space [GO:0005615]; nucleus [GO:0005634]	Cytokine activity [GO:0005125]; tumor necrosis factor receptor binding [GO:0005164]
**Q9MZV6**	Caspase-1 (AARPEHKS; AARPEHKSSDS)	CASP1	2.6553	1.08	Up	0.041404	Apoptotic process [GO:0006915]; positive regulation of cytokine production [GO:0001819]	AIM2 inflammasome complex [GO:0097169]; IPAF inflammasome complex [GO:0072557]	Cysteine-type endopeptidase activity [GO:0004197]
**P41683**	Interleukin-6 (ADKMEELIKY; AEKDGCFQSGFNQ)	IL6	2.6337	1.09	Up	0.041404	Acute-phase response [GO:0006953]; glucose homeostasis [GO:0042593]	Extracellular space [GO:0005615]; interleukin-6 receptor complex [GO:0005896]	Cytokine activity [GO:0005125]; interleukin-6 receptor binding [GO:0005138]
**Q2VPW6**	5,6-dihydroxyindole-2-carboxylic acid oxidase (DHICA oxidase; AACDQRVLIVR; AALSLVAVIFAGASCMIRA)	TYRP1	2.6042	1.06	Up	0.041404	Melanin biosynthetic process [GO:0042438]; melanocyte differentiation [GO:0030318]; melanosome organization [GO:0032438]	Melanosome [GO:0042470]; melanosome membrane [GO:0033162]	Metal ion binding [GO:0046872]; monooxygenase activity [GO:0004497]
**P46402**	Interferon γ (IFN-γ) (AFQLCIIL; AMFFKEIEE)	IFNG	2.5167	1.11	Up	0.047268	Adaptive immune response [GO:0002250]; astrocyte activation [GO:0048143]	Extracellular space [GO:0005615]	Cytokine activity [GO:0005125]; type II interferon receptor binding [GO:0005133]
**P01354**	Gastrin (AAYGWMDF; AAYGWMDFG)	GAST	3.5541	0.9	Down	0.016134	G protein-coupled receptor signaling pathway [GO:0007186]; response to food [GO:0032094]	Extracellular space [GO:0005615]	Hormone activity [GO:0005179]
**Q95M33**	Interleukin-18 (AAIPVDDCI;ACEKEKDL)	IL18	3.0154	0.93	Down	0.027959	Cytokine-mediated signaling pathway [GO:0019221]; immune response [GO:0006955]; inflammatory response [GO:0006954]	Cytosol [GO:0005829]; extracellular space [GO:0005615]	Cytokine activity [GO:0005125]; interleukin-18 receptor binding [GO:0045515]

Significant differences were calculated using the Wilcoxon rank-sum test with a threshold of FDR-adjusted *P* < .05. Functional categories were assigned based on LC–MS/MS identification and Gene Ontology (GO) map.

Proteins were classified based on their GO functional roles, including biological processes, cellular components, and molecular functions. Note: Key protein ids with significant dysregulation (FDR-adjusted *P* < .05) are highlighted in boldface.

Abbreviations: CTRL = healthy control group; FC = fold change; FCGS = feline chronic gingivostomatitis; FDR = false discovery rate; N/A = not applicable.

### Functional enrichment and interaction networks of candidate peptides

**Figure 4:**
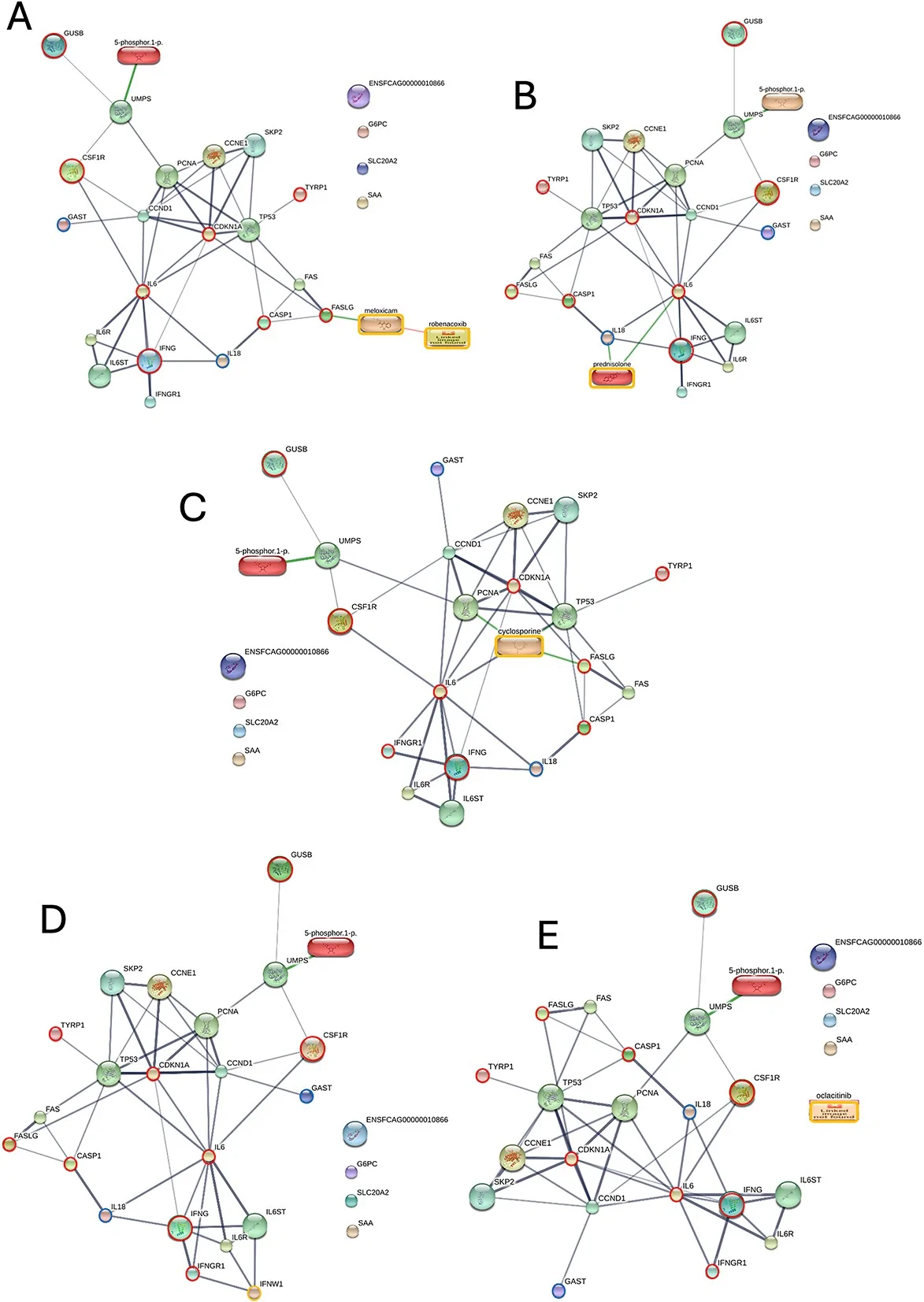
Protein–protein and protein–chemical interaction networks linked to therapeutic interventions. The networks show interactions among differentially expressed proteins and chemical compounds after treatment with NSAIDs (meloxicam and robenacoxib); (A), prednisolone (B), cyclosporine (C), interferon-ω (IFNW1; IFN-ω; D), and oclacitinib (E). Circular nodes with prominent outer ring highlights upregulated or downregulated proteins, and rectangular nodes signify drugs or chemicals. Edges indicate known or predicted interactions, with line thickness proportional to interaction confidence; thicker edges represent higher confidence interactions (confidence score > 0.7).

**Figure 5:**
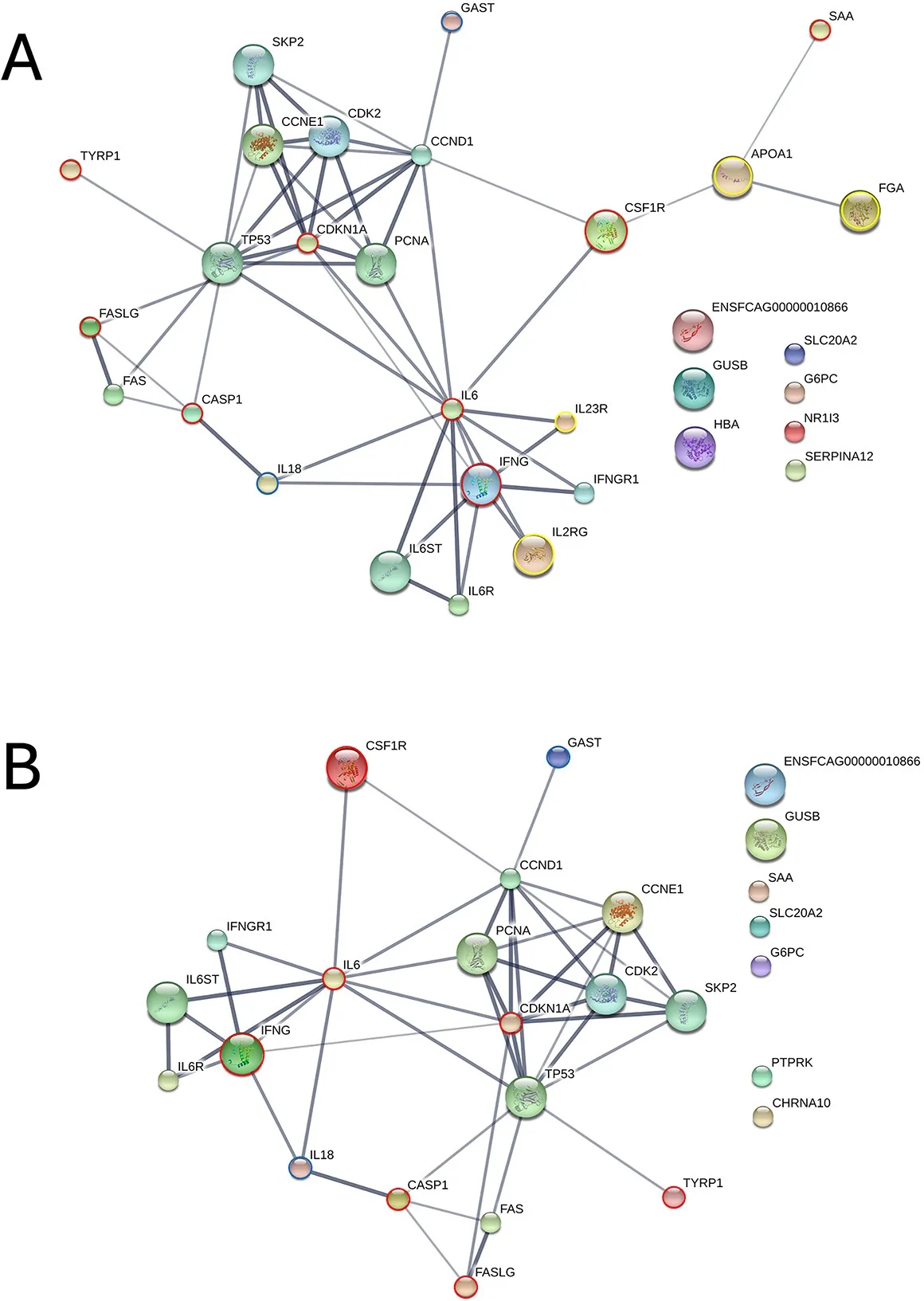
Protein–protein interaction networks of differentially expressed proteins. (A) Interaction network displaying core proteins (SAA, INHBA, G6PC1, SLC20A2(PiT-2), CDKN1A, PrP, CSF1R, GUSB, FASLG, CASP1, IL-6, TYRP1, IFNG/IFN-γ, GAST, and IL-18) connected to upregulated proteins (APOA1, FGA, HBA, IL2RG, IL23-R, NR1I3(CAR), and Serpin A12). (B) Network illustrating relationships between the same core protein set and downregulated proteins (PTPRK and CHRNA10). Notably, PTPRK and CHRNA10 did not show direct interactions with the main network and appear as isolated nodes. Circular nodes with prominent outer ring highlights indicate upregulated or downregulated proteins, standard circular nodes represent core proteins, and edge thickness reflects interaction confidence, with thicker edges indicating higher confidence interactions (confidence score > 0.7).

To characterize the systemic molecular landscape, upregulated proteins were mapped against established clinical treatments. Integrated networks identified 8 hallmark proteins (CDKN1A, CSF1R, GUSB, FASLG, CASP1, IL-6, TYRP1, and IFN-γ) that interacted with NSAIDs, prednisolone, cyclosporine, IFN-ω, and oclacitinib (Figure 4A-E), significantly enriching the JAK–STAT (FDR: 7.02 × 10^−8^) and the PI3K-Akt (FDR: 1.41 × 10^−7^) signaling pathways. Detailed topological descriptions and edge configurations for each drug-specific network are provided in the Supplementary material (Text S1; Tables S6-S10). Cross-compartment analysis linking serum findings to 9 previously identified salivary biomarkers (APOA1, FGA, HBA, IL2RG, IL-23R, CAR, Serpin A12, PTPRK, and CHRNA10) demonstrated coordinated regulation of systemic and local immune responses (Figure 5A). Core inflammatory hubs (IL-6, IFN-γ, and CDKN1A) were functionally linked to salivary IL2RG and IL-23R within the JAK–STAT (FDR: 6.19 × 10^−11^) and PI3K-Akt (FDR: 6.17 × 10^−10^) pathways (Text S2; Table S11). Conversely, PTPRK and CHRNA10 appeared as isolated nodes (Figure 5B), confirming that they are exclusively salivary biomarkers rather than systemic indicators.^15^

## Discussion

Cats were classified into either FCGS group or CTRL group based on characteristic oral lesions and clinical examination findings. A histologic examination was performed to exclude other oral disorders and showed that all FCGS biopsy samples featured severe inflammatory changes, reinforcing the clinical diagnosis.^4^^,^^12^ To investigate the potential role of retroviral infections and FHW in the development of FCGS, we used the SNAP Feline Triple test in both groups. Previous research has not established a definitive causal link between FeLV or FIV and the development of FCGS.^22^ Phenotypically, FeLV-positive cats often show unique oral signs, with fewer proliferative lesions but a higher tendency for ulcerative glossitis.^23^ Our results align with the low prevalence rates seen in previous studies. For example, a study of 60 FCGS-affected cats found FeLV and FIV positivity of 7% and 8%, respectively.^24^ Similarly, studies on calicivirus shedding found no significant link with FeLV, FIV, or feline herpesvirus.^25^ Despite FIV’s known ability to worsen local inflammation and influence the immune response, our data suggest these viruses more often are comorbidities rather than primary causes of the disease.^26^ In our study of 52 cats, FIV prevalence in the FCGS group was 11.67% (4/34) compared to 11.11% (2/18) in CTRL group. Likewise, FeLV prevalence was 8.82% (3/34) in FCGS cats and 5.56% (1/18) in CTRL cats. Fisher’s exact test showed that these differences were not significant (*P* = 1.1 for both FIV and FeLV), indicating no significant association between retroviral status and FCGS in this group. These findings are consistent with our previous salivary peptidomic study, which reported FeLV and FIV frequencies of 9.38% each in a 32-cat FCGS group.^15^ Remarkably, all subjects in our current study tested negative for FHW antigen, and only one cat (CTRL group) had FIV/FeLV coinfection. Although immune status and coinfections are often seen as risk factors for severe oral inflammation, our results support the idea that FCGS can develop independently of these systemic viral infections.^27^

Our serum analysis using MALDI-TOF MS identified clear, distinct clustering between the FCGS and CTRL groups, indicating that the systemic peptidome undergoes important changes during the disease process. These findings are consistent with known systemic immune dysregulation in FCGS, characterized by intense immune activation, neutrophilia, and marked increases in pro-inflammatory cytokines such as IL-6, TNF-α, and IFN-γ.^28–30^ The identified PMFs likely constitute a “peptide barcode” of this chronic inflammatory condition, characterized by a disrupted CD4/CD8 T-cell ratio and an increase in activated effector memory CD8+ T cells.^4^^,^^31^ Similar to our previous salivary studies, where PMFs were independent of viral status, these serum signatures support the dual nature of FCGS as both a local and systemic illness.^15^ The high level of profile consistency seen suggests that MALDI-TOF MS is a rapid, dependable, and non-invasive method to monitor the systemic environment in cats, providing a solid base for higher-resolution nano LC–MS/MS identification of specific candidate biomarkers.

The findings from serum peptidomic and network analyses provide a detailed molecular link between localized oral inflammation and systemic immune dysregulation in FCGS. The clear separation of serum peptide profiles between FCGS cats and healthy CTRL, as shown by LC–MS/MS and confirmed by 2D and 3D PLS-DA clustering, demonstrates that the disease is marked by high peptidomic variability and widespread systemic failure of homeostatic functions. The discovery of 15 serum proteins that are significantly altered, including 13 upregulated markers such as IL-6, IFN-γ, CASP1, and SAA, emphasizes the strongly pro-inflammatory nature of FCGS. These findings support previous transcriptomic research indicating that FCGS tissues are rich in immune- and inflammation-related pathways.^7^ Seven proteins (CDKN1A, CSF1R, GUSB, FASLG, CASP1, IL-6, TYRP1, and IFN-γ) interact and react to medications such as meloxicam, robenacoxib, prednisolone, IFN-ω, and cyclosporine. Although no direct drug-target binding was identified between oclacitinib and the 8 core proteins, the network exhibited distinct topological features, indicating that these markers are part of a coordinated downstream response to JAK inhibition. Functional analysis indicated that these proteins are involved in inflammatory pathways, with the JAK–STAT and PI3K-Akt signaling pathways predominant in both serum and salivary profiles, further supporting a unified signaling network driving the disease.^15^

The synthesis of current evidence suggests that *CDKN1A* (p21) serves as a pivotal molecular checkpoint that suppresses chronic inflammation by modulating the JAK–STAT and PI3K-Akt signaling axes. As a primary target of the p53 tumor suppressor gene *(TP53), CDKN1A* encodes p21, which induces cell cycle arrest and prevents over-activation of macrophages and production of pro-inflammatory cytokines.^32^^,^^33^ In inflammatory states such as rheumatoid arthritis (RA) or FCGS, the downregulation of *CDKN1A* potentially is involved in a loss of this inhibitory “brake,” facilitating activation of pSTAT-1 and the subsequent release of IL-6 and TNF-α.^34^ Conversely, the over-expression of *CDKN1A* has been shown to down-regulate these pro-inflammatory mediators while up-regulating the anti-inflammatory cytokine IL-10, thereby inhibiting proliferation of inflammatory cells.^34^^,^^35^

In our study, IL-6 emerged as a primary hub within the interaction networks, reflecting its role as a critical immunoregulatory cytokine that directs systemic inflammation through the JAK–STAT signaling axis.^36^ The observed upregulation of IL-6 is a likely driver of the clinicopathological hallmarks of FCGS, including polyclonal hypergammaglobulinemia and increased concentrations of acute phase proteins.^37^^,^^38^ The addition of IL-6 signaling exacerbates this dysregulation via separate route combinations. Although conventional IL-6 signaling largely stimulates the JAK–STAT3 pathway, IL-6 trans-signaling, a substantial cause of chronic disease, also recruits the PI3K–Akt and MEK–ERK pathways.^39^ This simultaneous activation is required for the induction of chemokines such as MCP-1, which perpetuates the recruitment of inflammatory cells into diseased tissues.^39^ Therefore, the dominance of IL-6 and the relative deficiency or functional isolation of CDKN1A identified in our serum peptidomic network provides a molecular rationale for the persistent, proliferative inflammation observed in FCGS. Specifically, we identified a significant upregulation of SAA in the serum of FCGS-afflicted cats. Interestingly, although, IL-6 is known to induce SAA, our network analysis indicated that SAA did not exhibit a high-confidence linkage to any of the therapeutic agents investigated, despite their association with the IL-6 hub. This finding suggests that, although SAA is a robust indicator of the systemic inflammatory state in FCGS, its modulation by current pharmacological interventions may be indirect or distinct from the primary cytokine inhibition pathways. Furthermore, IL-6’s capacity to inhibit apoptosis may provide a molecular basis for the proliferative nature of the lymphoplasmacytic mucosal lesions that characterize the disease.^40^ The concurrent upregulation of IFN-γ and the enrichment of IFN type I and II pathways strongly suggest a persistent, unresolved immune response to viral antigens, potentially stemming from chronic FCV infection.^7^^,^^41^ Interferon gamma is a known inducer of chemotactic molecules, such as CXCL10, which facilitate the trafficking of T and B lymphocytes into the oral mucosa, thereby perpetuating local tissue destruction.^42^ Our systemic findings strongly correspond with previous transcriptome investigations of oral mucosal tissues, which are mostly enriched with immune- and inflammation-related *IL6* genes, notably those regulated by NF-kB, JAK-STAT, IL-17, and interferon signaling pathways.^7^ Interestingly, our findings show that the same signaling pathways are detectable in the serum peptidome, despite earlier transcriptomic profiles of caudal oral mucosal swabs showing significant induction of the PI3K-Akt and SAPK/JNK signaling pathways, noting their modulation after therapeutic interventions such as full-mouth extractions or mesenchymal stem cell treatment.^6^ Notably, although the specific protein identities in our serum peptidomic analysis do not perfectly overlap with those previously identified by salivary profiling, the fundamental signaling architectures, including the JAK–STAT and PI3K–Akt pathways, remain consistent across both biofluids.^15^ The dominance of CDKN1A, IL-6, and IFN-γ within the serum provides clear evidence that the molecular disturbances of FCGS are not restricted to the oral cavity but represent a broader systemic dysregulation. This unified understanding of the JAK–STAT axis as a central driver of both local and systemic disease provides a rational foundation for the development of novel, targeted clinical applications, such as selective JAK inhibitors for the management of refractory FCGS cases.

Interestingly, our analysis identified a significant downregulation of serum IL-18, typically considered a pro-inflammatory cytokine.^43^ This systemic decrease, alongside upregulation of IL-6 and IFN-γ, may indicate that IL-18 is preferentially sequestered or consumed at sites of intense oral inflammation. Alternatively, it could suggest activation of compensatory mechanisms, such as production of IL-18 binding protein (IL-18BP), to neutralize circulating IL-18 and mitigate chronic systemic inflammatory damage.^44^ These findings emphasize that the systemic peptide profile in FCGS reflects a complex balance between pro-inflammatory signals and the host’s attempt at immune regulation.

The identification of CSF1R as a significantly upregulated node in our serum peptidomic analysis aligns with its established role as a primary regulator of mononuclear phagocytic proliferation, morphology, and chemotaxis within inflammatory foci.^45^ As a receptor tyrosine kinase, CSF1R mediates the pleiotropic effects of its ligand, CSF-1, through the phosphorylation of intracellular tyrosine residues that serve as docking sites for downstream signaling cascades.^46^ Crucially, aberrant CSF1R activation has been directly linked to the exacerbation of chronic inflammatory diseases and the promotion of malignant growth in T-cell lymphomas via activation of the PI3K/Akt/mTOR signaling axis.^47^ In the context of FCGS, the overexpression of CSF1R in serum likely reflects a systemic expansion and activation of the mononuclear phagocytic lineage, in which it may act in tandem with the PI3K/Akt pathway to perpetuate the aggressive tissue proliferation and immune dysregulation characteristic of the disease.

The identification of GUSB as a significantly upregulated node in our serum peptidomic analysis suggests a critical mechanism for the amplification of systemic inflammation in FCGS. Increased activity of this enzyme correlates with the recycling and release of inflammatory mediators within tissues, effectively fueling a pro-inflammatory feedback loop.^48^^,^^49^ By contributing to the increased production of IL-6, GUSB indirectly is associated with the overactivation of the JAK–STAT signaling pathway, which we identified as a dominant feature of the FCGS proteomic network. Consequently, the high confidence interaction between GUSB and inflammatory hubs in our study reinforces the potential of targeting this enzyme to attenuate the downstream transcription of inflammation-associated genes and systemic dysregulation.

The identification of CASP1 as a significantly upregulated node in our serum peptidomic analysis provides a critical link to the activation of the innate immune system and the progression of chronic systemic inflammation in FCGS. As a key proteolytic enzyme within the inflammasome complex, CASP1 cleaves pro-inflammatory cytokines into their biologically active forms, thereby driving the recruitment of inflammatory cells and exacerbating tissue damage.^50^ Bioinformatic evidence from other chronic inflammatory models indicates that increased CASP1 expression is strongly associated with the PI3K-Akt signaling pathway, as well as unfavorable prognoses in conditions characterized by persistent stimulus responses and extracellular region dysregulation.^51^

The identification of TYRP1 as a significantly upregulated node in our serum peptidomic analysis provides a novel link between systemic chronic inflammation and dysregulated biosynthetic pathways in FCGS. Tyrosine-related protein 1 is a critical enzyme in melanin synthesis, and its expression is tightly regulated by the PI3K/Akt signaling pathway, which serves as a key upstream activator of the transcription factor microphthalmia-associated transcription factor.^52^ The detection of TYRP1 in the serum of cats with FCGS is unusual because this enzyme usually is found inside melanosomes. Its presence likely indicates severe, chronic oral mucosal inflammation causing cell death and tissue lysis that release intracellular components into the systemic circulation. Chronic inflammation may also activate melanocytes, causing hyperpigmentation in oral lesions and possibly increasing TYRP1 concentrations. These mechanisms suggest TYRP1 could indicate the extent of mucosal damage and pigmentary changes in FCGS. Although traditionally associated with melanogenesis, the increase of TYRP1 in serum may reflect marked systemic control failure and oxidative stress characteristic of chronic disease states.^53^ Furthermore, the activation of the PI3K/Akt axis in response to inflammatory cytokines, which is a hallmark of the FCGS proteomic profile, suggests that TYRP1 upregulation is part of a broader systemic dysregulation of metabolic and signaling pathways. Consequently, the integration of TYRP1 into the core inflammatory network reinforces the evidence of widespread systemic involvement and emphasizes the convergent role of the PI3K/Akt pathway in driving the complex pathology of FCGS.

An unexpected finding in our serum analysis was the increased expression of PrP. Although it is most known for its function in the nervous system, PrP also is found on various immune cells, such as T-lymphocytes and macrophages, where it plays a role in cellular signaling and immune regulation during chronic inflammation.^54^^,^^55^ In FCGS, higher serum PrP concentrations may indicate ongoing immune activation or a response to persistent oxidative stress in the oral cavity and general circulation. Additional studies are needed to clarify how PrP specifically contributes to FCGS development.

The protein interaction network analysis connecting serum findings with local salivary proteins (eg, IL2RG, IL-23R) emphasizes the coordination between circulating mediators and oral pathology. Combining our novel serum peptidomic data with previous salivary profiling suggests a highly coordinated molecular link between the localized oral environment and systemic circulation in cats with FCGS.^15^ Although specific protein identities differ among biofluids, with saliva showing increases in APOA1, CAR, FGA, IL2RG, and IL-23R, and serum dominated by the IL-6/IFN-γ axis and the loss of the CDKN1A (p21) inhibitory brake, both compartments converge on the JAK–STAT and PI3K-Akt signaling pathways as primary drivers of the pathogenesis. The detection of HBA and Serpin A12 in saliva suggests a localized attempt to counteract oxidative stress via the PI3K–Akt pathway, a physiological challenge we now confirm as systemic, as evidenced by the serum upregulation of TYRP1 and the CSF1R/CASP1 amplification loop. This shared signaling framework at both local and systemic levels indicates that the intense mucosal inflammation and subsequent vicious cycle of tissue damage are maintained by a global dysregulation of these 2 key axes. As a result, the consistent detection of these pathways across different sample types strengthens the molecular rationale for using targeted immunomodulators, such as JAK–STAT or PI3K–Akt inhibitors, to offer a comprehensive therapeutic approach for refractory FCGS cases. Although some proteins, such as PTPRK and CHRNA10, are specifically downregulated in saliva, their lack of integration into the serum network emphasizes an important difference between local tissue expression and circulating profiles. This finding highlights the need for distinct biomarkers for monitoring local versus systemic disease. However, the 7-day medication washout period used in our study may be a limitation because certain long-acting drugs, such as corticosteroids or antibiotics, could have prolonged effects on the systemic peptidomic profile. Future longitudinal studies with longer washout periods or treatment-naive cohorts are warranted to further characterize the influence of prior medical management on these molecular signatures. Furthermore, we did not categorize cats based on viral load (specifically FCV) or disease severity. The cohort mainly included cats with severe, active FCGS before surgery to identify core systemic biomarkers. Although this approach highlights molecular markers of advanced disease, future research with ongoing viral load monitoring and clinical scores will improve the diagnostic and prognostic usefulness of these serum peptides. Additionally, the cohort’s single-center recruitment in Thailand and the lack of pedigreed breeds in the FCGS group may limit generalizability.

It is also important to recognize the methodological limitations of our study. Given its cross-sectional, observational design, the molecular signatures identified should be regarded as associations with the disease condition rather than as specific causal relationships. Moreover, although rigorous FDR corrections were implemented to decrease the likelihood of false positives in high-dimensional proteomics data, the sample size still may have been insufficient to definitively confirm pathway-level certainty. Although MALDI-TOF MS offers a rapid and cost-effective platform for serum profiling, our findings represent an initial discovery phase. To establish these peptide signatures as clinical biomarkers, further validation in larger, independent cohorts is needed. Future research should focus on determining optimal clinical thresholds and calculating diagnostic metrics, such as receiver operating characteristic (ROC) curves, sensitivity, and specificity to ensure the robustness and generalizability of the proposed diagnostic framework for FCGS. Consequently, our findings represent putative pathways that provide a critical molecular framework for FCGS and serve as a foundation for future large-scale longitudinal studies to validate these systemic biomarkers.

In conclusion, integrating serum peptidomic analysis with previous studies confirms that FCGS is a complex systemic syndrome driven by a unified signaling network. Our findings indicate that although the protein compositions of saliva and serum may differ, both compartments utilize the JAK–STAT and PI3K–Akt pathways as key drivers of chronic inflammation and tissue proliferation. Specifically, the systemic loss of the p53-CDKN1A (p21) inhibitory brake allows IL-6 and IFN-γ to dominate, creating a vicious cycle of immune imbalance that is further worsened by CSF1R-mediated macrophage activation and the enzymatic activity of CASP1 and β-glucuronidase. Additionally, the discovery of TYRP1 and the presence of HBA and Serpin A12 highlight clinically relevant oxidative stress and metabolic changes that characterize the disease both locally and systemically. These insights expand the clinical understanding of FCGS beyond being localized the oral cavity, providing a solid molecular foundation for using targeted immunomodulators such as JAK inhibitors or PI3K-Akt antagonists, especially in cases that do not respond to standard surgical treatment. Ultimately, this cross-compartment proteomic profiling advances our knowledge of the molecular pathogenesis of FCGS and offers a framework for personalizing therapeutic strategies, paving the way for more targeted treatments to improve patient outcomes.

## Supplementary Material

Supplementary_material_aalag169

## Data Availability

The MS/MS raw data and analysis files have been deposited with the ProteomeXchange Consortium (http://proteomecentral.proteomexchange.org) via the jPOST partner repository JPST004446 with the data set identifier PXD075136.

## Off-label antimicrobial declaration

Authors declare no off-label use of antimicrobials.

## Institutional animal care and use committee or other approval declaration

Approved by the Faculty of Veterinary Science, Mahidol University-Animal Care and Use Committee (MUVS-ACUC), Thailand (approval number MUVS-2023-10-64).

## Appendix

### Materials and methods

#### Animals and sampling

We employed a prospective case–control design comparing cats with FCGS to a healthy CTRL. The 34 cats in FCGS group aged 2-14 years, diagnosed with severe caudal stomatitis. The CTRL group comprised 18 healthy cats, aged 5-14 years. All cats underwent comprehensive initial health assessments, including blood testing, chemistry, parasite checks, and vaccination records. Inclusion for the CTRL group required the absence of caudal stomatitis and systemic illness, along with a CBC, serum biochemistry panel, and no evidence of severe periodontitis. For the FCGS group, inclusion criteria required the presence of gingivitis, stomatitis, and caudal mucositis, with no other systemic illness, and a normal serum biochemistry panel except for the common finding of hyperproteinemia and hyperglobulinemia.^16^ The severity of oral inflammation in FCGS cats was confirmed by histologic examination as previously described.^15^ All samples were obtained with the owners’ informed written consent.

To preserve serum integrity,. The cats were fasted for 6-8 h before the procedure. Blood was drawn from all sedated or anesthetized cats before any oral interventions, following a pre-surgery collection protocol. Serum samples (about 1-2 mL) were immediately stabilized with protease inhibitor cocktails to protect the endogenous peptides and stored at −80 °C for future analysis. The total protein concentration in the final serum supernatant was accurately measured using the modified Lowry assay.^17^

#### Histopathology

Biopsies of the oral mucosa were performed in both the FCGS and CTRL groups while the cats were under general anesthesia during a dental procedure. Using iris scissors, tissue samples were specifically collected from the caudal region, lateral to the palatoglossal folds, to ensure capture of the most representative lesions in the FCGS-affected animals.^15^ These samples, obtained from visibly affected mucosa in all FCGS subjects, then were processed for further histological examination.

#### *Immunoassay for feline immunodeficiency virus antibodies, feline leukemia virus antigen, and* Dirofilaria immitis *antigen*

We compared the prevalence of FIV and FeLV between groups using χ-square or Fisher’s exact tests. Fisher’s test was chosen for small sample sizes (expected frequency < 5) to ensure accuracy. A *P*-value < .05 was established as the threshold for statistical significance.

#### Serum peptide profiling by mass spectrometry

*Cluster analysis by matrix-assisted laser desorption/ionization-time of flight mass spectrometry* (*MALDI-TOF MS)*

Serum samples were analyzed using MALDI-TOF MS. This mixture of serum peptides and matrix solution containing CHCA. was then directly applied onto a steel target plate (MTP 384 ground steel, JEOL, Japan). To ensure high analytical accuracy and reduce sample preparation bias, 8 replicates were prepared for each sample. Mass spectra were acquired using a JMS-S3000 SpiralTOF™-plus 2.0 in linear positive mode. The analyzed mass range was 1000-6000 Da. Each spectrum was generated from 500 laser shots at a 50-Hz frequency.^14^ External calibration was carefully performed using the Proteomass™ Peptide & Protein MALDI MS calibration kit (Sigma Aldrich, St. Louis, MO, USA).

The raw spectra obtained were processed and normalized using msTornado Analysis version 1.15. Peptide patterns were examined with statistical software, resulting in PMFs and PLS-DA scores. The main goal was to determine if the peptide profiles of FCGS cats and healthy controls formed separate clusters. All raw spectra underwent hierarchical clustering analysis using the Sartorius Stedim Data Analytics’ prediction engine (SIMCA®). The PLS-DA was conducted on 8 replicates per cat. Samples showing unusual profiles were excluded from the final PLS-DA plots. To ensure internal consistency and validation, 4 replicates from each group were pooled, and thirty-two replicates of these pooled samples were re-analyzed by MALDI-TOF MS to verify group uniformity.

*Peptide identification by nanoscale liquid chromatography–tandem mass spectrometry (nano LC–MS/MS)*

Peptides were chromatographically separated using an Ultimate 3000 Nano/Capillary LC System (Thermo Scientific, UK). A 100 nanogram of the peptide solution underwent enrichment using a μ-Precolumn (300 μm i.d. × 5 mm) packed with C18 Pepmap 100. Separation then was carried out on a C18 column (75 μm i.d. × 15 cm) packed with Acclaim PepMap RSLC C18 using nanoViper technology (Thermo Scientific, UK). The column temperature was maintained at 60°C in a temperature-controlled oven. Peptides were eluted over 30 min at a constant flow rate of 0.30 μL/min. Using a gradient of Solvent B (0.1% formic acid in 80% acetonitrile) ranging from 5% to 55%, with Solvent A (0.1% formic acid in water) as the aqueous phase. The LC system was coupled to a ZenoTOF 7600 mass spectrometer (SCIEX, Framingham, MA, USA). Source parameters were configured for positive polarity with a spray voltage of 3300 V and a source temperature of 200 °C. Ion source gas 1, curtain gas, and collisionally activated dissociation gas were set at 8, 35, and 7 psi, respectively.

The instrument operated in DDA mode with a total cycle time of 3.0 s. For MS/MS analysis, the 60 most abundant precursor ions from MS1 scans (intensity threshold > 150 cps) were selected. These ions were dynamically excluded for 12 s after 2 MS/MS samplings. The MS2 spectra were acquired in the 100 to 1800 m/z range with a 50 ms accumulation time. The Zeno trap was activated, and its threshold was set to 150 000 cps, summing over 8 time bins. The collision energy parameters for the ZenoTOF system included a declustering potential of 80 V, with no spread applied to the declustering potential (DP) or capillary electrophoresis-mass spectrometry (CE). The LC–MS analysis of each sample was done in triplicate.

#### Bioinformatics and statistical analysis

Protein and peptide quantification within individual samples was primarily managed using MaxQuant (version 2.5.0.0).^11^ The Andromeda search engine facilitated the matching of tandem mass spectrometry (MS/MS) spectra against the target proteome, referenced by a FASTA file containing the *F. catus* proteome downloaded from UniProt on November 2, 2025. Label-free quantification was implemented using standard parameters,^3^ nonspecific digestion mode, and employing a primary search mass tolerance of 0.6 Da.^4^ Methionine oxidation and protein N-terminus acetylation were specified as variable modifications.^5^ To ensure data reliability for subsequent analysis, proteins were required to be identified by a minimum of 2 peptides, one of which had to be unique, and peptides themselves required a minimum length of 7 amino acids.^6^ Quality control included applying a 1% protein FDR, estimated using reversed search sequences, and limiting modifications to a maximum of 5 per peptide.^7^

Statistical and functional interpretations followed this quantification. Multivariate analysis, presented via PLS-DA plots, and univariate analysis, displayed through a Manhattan plot, were performed using MetaboAnalyst 6.0 (http://www.metaboanalyst.ca).^18^ Peptides were deemed significantly altered if they concurrently met stringent criteria: FDR–adjusted *P*-value < .05.

#### Pathway and interaction analysis

The functional investigation of the identified candidate peptides focused on protein interaction networks and biological pathways. Protein–protein and protein–chemical interaction networks were explored using the “Stitch EMBL” database (accessible at “https://stitch.embl.de”), which was accessed on November 8, 2025.^19^ This analysis was complemented by functional enrichment analysis using ShinyGo v. 0. 85, leveraging the *F. catus* database.^20^ The following parameters were universally applied across all enrichment analyses, including categories such as biological process, molecular function, and cellular component: FDR*—adjusted P-*value (*Q*) < .05 was set as the significance threshold. Further filtering involved restricting the pathway size to between 2 and 5000. The “remove redundancy” and “abbreviate pathway names” options were enabled to improve the clarity and specificity of the results. Additionally, KEGG pathway analysis was used to formally examine protein interactions within known biological cascades. This analysis specifically investigated the interaction profiles of the proteins with current therapeutic agents used to treat FCGS, including NSAIDs such as meloxicam and robenacoxib, steroids like prednisolone, and immunomodulators like IFN-ω, cyclosporin, and oclacitinib. Furthermore, dedicated protein–protein interaction networks were analyzed for 9 specific proteins—7 previously reported as upregulated and 2 as downregulated in the saliva of FCGS cats.^15^ The 7 upregulated proteins investigated included apolipoprotein A1 (APOA1), fibrinogen α chain (FGA), hemoglobin subunit α (HBA), interleukin 2 receptor γ (IL2RG), interleukin 23 receptor (IL23R), nuclear receptor subfamily 1 group I member 3 (NR1I3), and serpin peptidase inhibitor clade A (α-1 antiproteinase, antitrypsin) member 12 (SERPINA 12). The 2 downregulated proteins were protein tyrosine phosphatase (PTPRK) and cholinergic receptor, nicotinic α 10 subunit (CHRNA10). This detailed analysis aimed to precisely delineate the signaling pathways affected in FCGS pathogenesis.

## Abbreviations

FCGS: feline chronic gingivostomatitis
JAK/STAT: Janus kinase/signal transducers and activators of transcription
KEGG: Kyoto Encyclopedia of Genes and Genomes
LC–MS/MS: liquid chromatography–tandem mass spectrometry
MALDI-TOF: matrix-assisted laser desorption/ionization-time-of-flight
MS: mass spectrometry
PI3K/Akt: phosphatidylinositol 3-kinase (PI3K)/protein kinase B (AKT)
PMF: peptide mass fingerprint

## References

[1] Druet I, Hennet P. Relationship between feline calicivirus load, oral lesions, and outcome in feline chronic gingivostomatitis (caudal stomatitis): retrospective study in 104 cats. Front Vet Sci. 2017;4:209. 10.3389/fvets.2017.00209 PMC5724031 29270412

[2] Soltero-Rivera M, Goldschmidt S, Arzi B. Feline chronic gingivostomatitis: current concepts in clinical management. J Feline Med Surg. 2023;25:1-16. 10.1177/1098612X231186834 PMC10811996 37548475

[3] Harley R, Gruffydd-Jones TJ, Day MJ. Immunohistochemical characterization of oral mucosal lesions in cats with chronic gingivostomatitis. J Comp Pathol. 2011;144:239-250. 10.1016/j.jcpa.2010.09.173 21084098

[4] Vapniarsky N, Simpson DL, Arzi B, et al. Histological, immunological, and genetic analysis of feline chronic gingivostomatitis. Front Vet Sci. 2020;7:310. 10.3389/fvets.2020.00310 PMC7283503 32582783

[5] Lee D . Bin, Verstraete FJM, Arzi B. An update on feline chronic gingivostomatitis. Vet Clin North Am Small Anim Pract. 2020;50:973-982. 10.1016/j.cvsm.2020.04.002 32360016 PMC7194110

[6] Soltero-Rivera M, Shaw C, Arzi B, Lommer M, Weimer B. Feline chronic gingivostomatitis diagnosis and treatment through transcriptomic insights. Pathogens. 2024;13:192. 10.3390/pathogens13030192 38535535 PMC10974286

[7] Peralta S, Grenier JK, Webb SM, Miller AD, Miranda IC, Parker JSL. Transcriptomic signatures of feline chronic gingivostomatitis are influenced by upregulated IL6. Sci Rep. 2023;13:13437. 10.1038/s41598-023-40679-4 37596310 PMC10439118

[8] Harley R, Gruffydd-Jones TJ, Day MJ. Determination of salivary and serum immunoglobulin concentrations in the cat. Vet Immunol Immunopathol. 1998;65:99-112. 10.1016/S0165-2427(98)00146-9 9839866

[9] Mestrinho LA, Rosa R, Ramalho P, et al. A pilot study to evaluate the serum alpha-1 acid glycoprotein response in cats suffering from feline chronic gingivostomatitis. BMC Vet Res. 2020;16:390. 10.1186/s12917-020-02590-2 33059691 PMC7558631

[10] Kouki MI, Papadimitriou SA, Psalla D, Kolokotronis A, Rallis TS. Chronic gingivostomatitis with esophagitis in cats. J Vet Intern Med. 2017;31:1673-1679. 10.1111/jvim.14850 28960466 PMC5697197

[11] Bellei E, Dalla F, Masetti L, Pisoni L, Joechler M. Surgical therapy in chronic feline gingivostomatitis (FCGS). Vet Res Commun. 2008;32:231-234. 10.1007/s11259-008-9153-8 18685967

[12] Jennings MW, Lewis JR, Soltero-Rivera MM, Brown DC, Reiter AM. Effect of tooth extraction on stomatitis in cats: 95 cases (2000–2013). J Am Vet Med Assoc. 2015;246:654-660. 10.2460/javma.246.6.654 25719848

[13] Kycko A, Reichert M. Proteomics in the search for biomarkers of animal cancer. Curr Protein Pept Sci. 2014;15:36-44. 10.2174/1389203715666140221110945 24555898

[14] Ploypetch S, Jaresitthikunchai J, Phaonakrop N, et al. Utilizing MALDI-TOF MS and LC-MS/MS to access serum peptidome-based biomarkers in canine oral tumors. Sci Rep. 2022;12:21641. 10.1038/s41598-022-26132-y 36517562 PMC9750994

[15] Ploypetch S, Pornthummawat A, Roytrakul S, et al. Salivary peptidomic profiling of chronic gingivostomatitis in cats by matrix-assisted laser desorption/ionization-time-of-flight mass spectrometry and nanoscale liquid chromatography-tandem mass spectrometry. J Vet Intern Med. 2025;39:e17247. 10.1111/jvim.17247 PMC11627522 39576047

[16] Tyanova S, Temu T, Cox J. The MaxQuant computational platform for mass spectrometry-based shotgun proteomics. Nat Protoc. 2016;11:2301-2319. 10.1038/nprot.2016.136 27809316

[17] UniProt Consortium T . UniProt: the universal protein knowledgebase. Nucleic Acids Res. 2018;46:2699-2699. 10.1093/nar/gky092 29425356 PMC5861450

[18] Ewald JD, Zhou G, Lu Y, et al. Web-based multi-omics integration using the analyst software suite. Nat Protoc. 2024;19:1467–1497. 10.1038/s41596-023-00950-4 38355833

[19] Szklarczyk D, Santos A, von Mering C, Jensen LJ, Bork P, Kuhn M. STITCH 5: augmenting protein–chemical interaction networks with tissue and affinity data. Nucleic Acids Res. 2016;44:D380-D384. 10.1093/nar/gkv1277 26590256 PMC4702904

[20] Ge SX, Jung D, Yao R. ShinyGO: a graphical gene-set enrichment tool for animals and plants. Bioinformatics. 2020;36:2628-2629. 10.1093/bioinformatics/btz931 31882993 PMC7178415

[21] Sun Y, Liu N, Guan X, Wu H, Sun Z, Zeng H. Immunosuppression induced by chronic inflammation and the progression to oral squamous cell carcinoma. Mediators Inflamm. 2016;2016:1-12. 10.1155/2016/5715719 PMC5178366 28053372

[22] Tenorio AP, Franti CE, Madewell BR, Pedersen NC. Chronic oral infections of cats and their relationship to persistent oral carriage of feline calici-, immunodeficiency, or leukemia viruses. Vet Immunol Immunopathol. 1991;29:1-14. 10.1016/0165-2427(91)90048-H 1659031

[23] Silva M, Fernandes M, Fialho M, Mestrinho L. A case series analysis of dental extractions’ outcome in cats with chronic gingivostomatitis carrying retroviral disease. Animals. 2021;11:3306. 10.3390/ani11113306 34828037 PMC8614259

[24] Bellows J . Oropharyngeal inflammation. In: Bellows J, (ed.) Feline Dentistry. 2nd ed. Wiley-Blackwell; 2022:253-324.

[25] Greenfield B . Chronic feline gingivostomatitis: proven therapeutic approaches and new treatment options. Todays Vet Pract. 2017;7:26-38. Accessed Mar 2, 2026. https://todaysveterinarypractice.com/dentistry/chronic-feline-gingivostomatitis-proven-therapeutic-approaches-new-treatment-optionsce-article/

[26] Kornya MR, Little SE, Scherk MA, Sears WC, Bienzle D. Association between oral health status and retrovirus test results in cats. J Am Vet Med Assoc. 2014;245:916-922. 10.2460/javma.245.8.916 25285933

[27] Sánchez-Vallejo M, Vélez-Velásquez P, Correa-Valencia NM. Feline chronic gingivostomatitis: a thorough systematic review of associated factors. J Feline Med Surg. 2025;27:1-8. 10.1177/1098612X241310590 PMC12035028 40231602

[28] Bai J, Jiang L, Lin M, Zeng X, Wang Z, Chen Q. Association of Polymorphisms in the tumor necrosis factor-α and interleukin-10 genes with oral lichen planus: a study in a Chinese cohort with Han ethnicity. J Interferon Cytokine Res. 2009;29:381-388. 10.1089/jir.2008.0089 19450147

[29] Taghavi Zenouz A, Pouralibaba F, Babaloo Z, Mehdipour M, Jamali Z. Evaluation of serum TNF-α and TGF-β in patients with oral lichen planus. J Dent Res Dent Clin Dent Prospects. 2012;6:143-147. 10.5681/joddd.2012.029 23277861 PMC3529928

[30] Lopes RS, Carvalho PP, Pires MA, Rodrigues-Santos P, Costa E, Requicha JF. Local and systemic immunological response in feline chronic gingivostomatitis: a critical review. Front Immunol. 2025;16:1572631. 10.3389/fimmu.2025.1572631 41019092 PMC12460127

[31] Arzi B, Mills-Ko E, Verstraete FJM, et al. Therapeutic efficacy of fresh, autologous mesenchymal stem cells for severe refractory gingivostomatitis in cats. Stem Cells Transl Med. 2016;5:75-86. 10.5966/sctm.2015-0127 26582907 PMC4704876

[32] Saiz-Ladera C, Lara MF, Garín M, et al. p21 suppresses inflammation and tumorigenesis on pRB-deficient stratified epithelia. Oncogene. 2014;33:4599-4612. 10.1038/onc.2013.417 24121270 PMC4913869

[33] Mavers M, Cuda CM, Misharin AV, et al. Cyclin-dependent kinase inhibitor p21, via its C-terminal domain, is essential for resolution of murine inflammatory arthritis. Arthritis Rheum. 2012;64:141-152. 10.1002/art.33311 21898359 PMC3253189

[34] Gang X, Xu H, Si L, et al. Treatment effect of CDKN1A on rheumatoid arthritis by mediating proliferation and invasion of fibroblast-like synoviocytes cells. Clin Exp Immunol. 2018;194:220-230. 10.1111/cei.13161 29920650 PMC6194335

[35] Gamboni F, Anderson C, Mitra S, et al. Hypertonic saline primes activation of the p53–p21 signaling axis in human small airway epithelial cells that prevents inflammation induced by pro-inflammatory cytokines. J Proteome Res. 2016;15:3813-3826. 10.1021/acs.jproteome.6b00602 27529569 PMC5811188

[36] Heinrich PC, Behrmann I, Müller-Newen G, Schaper F, Graeve L. Interleukin-6-type cytokine signalling through the gp130/jak/STAT pathway. Biochem J. 1998;334:297-314. 10.1042/bj3340297 9716487 PMC1219691

[37] Rossi G . Acute phase proteins in cats: diagnostic and prognostic role, future directions, and analytical challenges. Vet Clin Pathol. 2023;52:37-49. 10.1111/vcp.13238 36740231

[38] Tetlow ER, Wentlent LA, Carney PC, et al. Serum protein alterations characterize feline chronic gingivostomatitis: a case-control study of electrophoresis, immunoglobulin, and cytokine profiles. Am J Vet Res. 2026;87:1-10. 10.2460/ajvr.26.02.0052 41946374

[39] Zegeye MM, Lindkvist M, Fälker K, et al. Activation of the JAK/STAT3 and PI3K/AKT pathways are crucial for IL-6 trans-signaling-mediated pro-inflammatory response in human vascular endothelial cells. Cell Commun Signal. 2018;16:55. 10.1186/s12964-018-0268-4 30185178 PMC6125866

[40] Xu G, Zhang Y, Zhang L, Ren G, Shi Y. The role of IL-6 in inhibition of lymphocyte apoptosis by mesenchymal stem cells. Biochem Biophys Res Commun. 2007;361:745-750. 10.1016/j.bbrc.2007.07.052 17678624 PMC2699935

[41] Wei Y, Zeng Q, Gou H, Bao S. Update on feline calicivirus: viral evolution, pathogenesis, epidemiology, prevention and control. Front Microbiol. 2024;15:1388420. 10.3389/fmicb.2024.1388420 PMC11096512 38756726

[42] Marshall A, Celentano A, Cirillo N, McCullough M, Porter S. Tissue-specific regulation of CXCL9/10/11 chemokines in keratinocytes: implications for oral inflammatory disease. PloS One. 2017;12:e0172821. 10.1371/journal.pone.0172821 28253295 PMC5333845

[43] Sedimbi SK, Hägglöf T, Karlsson MCI. IL-18 in inflammatory and autoimmune disease. Cell Mol Life Sci. 2013;70:4795-4808. 10.1007/s00018-013-1425-y 23892891 PMC11113411

[44] Ihim SA, Abubakar SD, Zian Z, et al. Interleukin-18 cytokine in immunity, inflammation, and autoimmunity: biological role in induction, regulation, and treatment. Front Immunol. 2022;13:919973. 10.3389/fimmu.2022.919973 PMC9410767 36032110

[45] Chitu V, Stanley ER. Colony-stimulating factor-1 in immunity and inflammation. Curr Opin Immunol. 2006;18:39-48. 10.1016/j.coi.2005.11.006 16337366

[46] Pixley FJ, Stanley ER. CSF-1 regulation of the wandering macrophage: complexity in action. Trends Cell Biol. 2004;14:628-638. 10.1016/j.tcb.2004.09.016 15519852

[47] Murga-Zamalloa C, Rolland DCM, Polk A, et al. Colony-stimulating factor 1 receptor (CSF1R) activates AKT/mTOR Signaling and promotes T-cell lymphoma viability. Clin Cancer Res. 2020;26:690-703. 10.1158/1078-0432.CCR-19-1486 31636099 PMC7002219

[48] Al-Khayri JM, Sahana GR, Nagella P, Joseph BV, Alessa FM, Al-Mssallem MQ. Flavonoids as potential anti-inflammatory molecules: a review. Molecules. 2022;27:2901. 10.3390/molecules27092901 35566252 PMC9100260

[49] Maleki SJ, Crespo JF, Cabanillas B. Anti-inflammatory effects of flavonoids. Food Chem. 2019;299:125124. 10.1016/j.foodchem.2019.125124 31288163

[50] Guo H, Callaway JB, Ting JPY. Inflammasomes: mechanism of action, role in disease, and therapeutics. Nat Med. 2015;21:677-687. 10.1038/nm.3893 26121197 PMC4519035

[51] Li Y, Du L, Meng L, Lv C, Tian X. High expression of CASP1 induces atherosclerosis. Medicine. 2024;103:e37616. 10.1097/MD.0000000000037616 38640260 PMC11030018

[52] Gelmi MC, Houtzagers LE, Strub T, Krossa I, Jager MJ. MITF in normal melanocytes, cutaneous and uveal melanoma: a delicate balance. Int J Mol Sci. 2022;23:6001. 10.3390/ijms23116001 35682684 PMC9181002

[53] Liu T, Xi T, Dong X, Xu D. Research progress on pathogenesis of skin pigmentation in chronic liver disease. Biomol Biomed. 2024;25:1218-1232. 10.17305/bb.2024.11085 PMC12042674 39689154

[54] Linden R . The biological function of the prion protein: a cell surface scaffold of signaling modules. Front Mol Neurosci. 2017;10:77. 10.3389/fnmol.2017.00077 PMC5357658 28373833

[55] Castle AR, Gill AC. Physiological functions of the cellular prion protein. Front Mol Biosci. 2017;4:19. 10.3389/fmolb.2017.00019 PMC5382174 28428956

