# Supplementary material for: Serum peptidomic profiling identifies systemic JAK–STAT and PI3K–Akt pathway dysregulation in cats with chronic gingivostomatitis
Source: J Vet Intern Med. 2026 Aug 25;40(4):aalag169. doi: 10.1093/jvimsj/aalag169 (PMC13505864; doi:10.1093/jvimsj/aalag169)
Supplement: Supplementary file 1 [file supplementary_material_aalag169.zip › suppl.docx]

**SUPPLEMENTARY MATERIALS**

**Supplementary Table 1.** List of peptide sequences, peptide intensity, protein names with biological process, cellular component, and molecular function detected from nanoLC-MS/MS measurement of the samples.

**Supplementary Table 2.** Demonstrates all biological process analyses from a list of protein names identified by nanoLC-MS/MS.

**Supplementary Table 3.** Demonstrates all cellular component analyses from a list of protein names identified by nanoLC-MS/MS.

**Supplementary Table 4.** Demonstrates all molecular function analyses from a list of protein names identified by nanoLC-MS/MS.

**Supplementary Table 5.** Demonstrates all Kyoto Encyclopedia of Genes and Genomes (KEGG) pathway analyses from a list of protein names identified by nanoLC-MS/MS.

**Supplementary Table 6.** Pathway analyses demonstrating protein and non-steroidal anti-inflammatory drug (meloxicam and robenacoxib) interaction networks using the Kyoto Encyclopedia of Genes and Genomes (KEGG) database.

**Supplementary Table 7.** Pathway analyses demonstrating protein-prednisolone interaction networks using the Kyoto Encyclopedia of Genes and Genomes (KEGG) database.

**Supplementary Table 8.** Pathway analyses demonstrating protein-cyclosporin interaction networks using the Kyoto Encyclopedia of Genes and Genomes (KEGG) database.

**Supplementary Table 9.** Pathway analyses demonstrating protein-interferon omega interaction networks using the Kyoto Encyclopedia of Genes and Genomes (KEGG) database.

**Supplementary Table 10.** Pathway analyses demonstrating protein-oclacitinib interaction networks using the Kyoto Encyclopedia of Genes and Genomes (KEGG) database.

**Supplementary Table 11.** Pathway analyses demonstrating protein interaction networks involving upregulated salivary proteins (APOA1, FGA, HBA, IL2RG, IL23-R, NR1I3 (CAR), and Serpin A12) using the Kyoto Encyclopedia of Genes and Genomes (KEGG) database.

**Supplementary Table 11.** Pathway analyses demonstrating protein interaction networks involving downregulated salivary proteins (PTPRK and CHRNA10) using the Kyoto Encyclopedia of Genes and Genomes (KEGG) database.
