# Supplementary material for: Serum peptidomic profiling identifies systemic JAK–STAT and PI3K–Akt pathway dysregulation in cats with chronic gingivostomatitis
Source: J Vet Intern Med. 2026 Aug 25;40(4):aalag169. doi: 10.1093/jvimsj/aalag169 (PMC13505864; doi:10.1093/jvimsj/aalag169)
Supplement: Supplementary file 1 [file supplementary_material_aalag169.zip › SUPPORTING INFORMATION.docx]

**SUPPORTING INFORMATION**

*Manuscript***:** serum peptidomic profiling reveals systemic JAK-STAT and PI3K-Akt pathway dysregulation in feline chronic gingivostomatitis

Supplement to the result section 3.5

Extended topological analysis of drug-chemical networks

*Text S1*: In the NSAID-associated network (meloxicam and robenacoxib), we observed a dense interaction cluster primarily focused on inflammation and cell cycle regulation (Figure 4A). IL-6 and CDKN1A served as the highest interaction hubs (edge confidence >0.9), bridging the PI3K-Akt signaling mediators with inflammatory cascades such as the TNF signaling pathway (FDR: 9.16 x 10^-3^). The additional connectivity of FASLG and CSF1R (edge confidence >0.7) further suggests that NSAIDs in FCGS potentially modulate apoptotic and inflammatory signaling through these pathways (Table S6). The prednisolone network exhibited a robust and widespread immunomodulatory profile (Figure 4B). The high-confidence interactions involving the CDKN1A-TP53 axis and PCNA reflect the potent effect of corticosteroids on the PI3K-Akt pathway and cell cycle arrest (Table S7). Furthermore, prednisolone demonstrates regulatory patterns linked to the IL-17 signaling pathway (FDR: 6.13 x 10^-3^) via IL-6 and IFN-γ, providing a molecular basis for its efficacy in controlling the intense mucosal inflammation characteristic of the disease. Within the cyclosporine network, the drug was directly incorporated into a central signaling hub, demonstrating high-confidence interactions (>0.7) with CDKN1A and downstream mediators IL-6 and IFN-γ (Figure 4C). This topology indicates that cyclosporine’s therapeutic mechanism in FCGS is heavily reliant on the inhibition of the JAK-STAT and PI3K-Akt signaling axes, effectively suppressing T-cell activation and cytokine production (Table S8). The IFN-ω network was uniquely characterized by an enhancement of antiviral and immunomodulatory signaling (Figure 4D). While maintaining a core architecture similar to other treatments, interaction strengths between interferon receptor 1 (IFNGR1) and downstream components were notably increased. This reflects targeted modulation of the JAK-STAT signaling pathway, specifically through upregulation of IFN-γ-associated pathways, which likely addresses the chronic viral antigenic stimulation suspected in FCGS pathogenesis (Table S9). The oclacitinib-associated network (Figure 4E) exhibited distinct topological features compared with other treatment networks, characterized by altered connectivity among inflammatory mediators and signaling nodes, consistent with selective modulation of Janus kinase-dependent pathways (Table S10).

*Text S2*: Protein-protein interaction network analysis identified a core set of differentially expressed proteins, including SAA, INHBA, G6PC1, PiT-2, CDKN1A, PrP, CSF1R, GUSB, FASLG, CASP1, IL-6, TYRP1, IFN-γ, GAST, and IL-18, that formed the central network structure. They were conducted to correlate serum findings with nine previously identified salivary biomarkers.^15^ The seven upregulated proteins investigated included apolipoprotein A1 (APOA1), fibrinogen alpha chain (FGA), hemoglobin subunit alpha (HBA), interleukin 2 receptor gamma (IL2RG), interleukin 23 receptor (IL-23R), nuclear receptor subfamily 1 group I member 3/Constitutive androstane receptor (CAR), and serpin peptidase inhibitor clade A (alpha-1 antiproteinase, antitrypsin) member 12 (Serpin A12). The two downregulated proteins were protein tyrosine phosphatase (PTPRK) and cholinergic receptor, nicotinic alpha 10 subunit (CHRNA10). This detailed analysis aimed to clearly define the signaling pathways affected in FCGS development. In Figure 5A, this core network connected to several upregulated proteins, including APOA1, FGA, HBA, IL2RG, IL23-R, CAR, and Serpin A12, indicating coordinated regulation of inflammatory, metabolic, and immune pathways. Functional enrichment analysis further clarifies this connection, as several linked proteins, especially IL2RG and IL-23R, are part of the core inflammatory hubs (IL-6, IFN-γ, and CDKN1A) within the JAK-STAT signaling pathway (FDR: 6.19 x 10^-11^) and the PI3K-Akt signaling pathway (FDR: 6.17 x 10^-10^). Additionally, these proteins are important in cytokine-cytokine receptor interactions (FDR: 3.16 x 10^-14^) and specific inflammatory pathways, such as the Inflammatory Bowel Disease pathway (FDR: 7.24 x 10^-10^), highlighting their biological importance in the overall systemic context of FCGS. These interactions indicate that systemic inflammation in FCGS is driven by a unified signaling network involving both local and circulating immune mediators (Table S11).
